# Avian influenza A (H7N9) virus: from low pathogenic to highly pathogenic

**DOI:** 10.1007/s11684-020-0814-5

**Published:** 2021-04-16

**Authors:** William J. Liu, Haixia Xiao, Lianpan Dai, Di Liu, Jianjun Chen, Xiaopeng Qi, Yuhai Bi, Yi Shi, George F. Gao, Yingxia Liu

**Affiliations:** 1 Shenzhen Key Laboratory of Pathogen and Immunity, Shenzhen Third People’s Hospital, Shenzhen, 518114 China; 2 National Institute for Viral Disease Control and Prevention, Chinese Center for Disease Control and Prevention, Beijing, 102206 China; 3 Laboratory of Protein Engineering and Vaccines, Tianjin Institute of Industrial Biotechnology, Chinese Academy of Sciences (CAS), Tianjin, 300308 China; 4 CAS Key Laboratory of Pathogenic Microbiology and Immunology, Institute of Microbiology, Chinese Academy of Sciences, Beijing, 100101 China; 5 CAS Key Laboratory of Special Pathogens and Biosafety, Chinese Academy of Sciences, Wuhan, 430071 China; 6 National Virus Resource Center, Chinese Academy of Sciences, Wuhan, 430071 China; 7 University of Chinese Academy Sciences, Beijing, 100049 China; 8 Center for Influenza Research and Early Warning, Chinese Academy of Sciences, Beijing, 100101 China; 9 Chinese Center for Disease Control and Prevention, Beijing, 102206 China

**Keywords:** H7N9, HPAIV, epidemiology, clinical features, pathogenesis, hemagglutinin, immunity, vaccine

## Abstract

The avian influenza A (H7N9) virus is a zoonotic virus that is closely associated with live poultry markets. It has caused infections in humans in China since 2013. Five waves of the H7N9 influenza epidemic occurred in China between March 2013 and September 2017. H7N9 with low-pathogenicity dominated in the first four waves, whereas highly pathogenic H7N9 influenza emerged in poultry and spread to humans during the fifth wave, causing wide concern. Specialists and officials from China and other countries responded quickly, controlled the epidemic well thus far, and characterized the virus by using new technologies and surveillance tools that were made possible by their preparedness efforts. Here, we review the characteristics of the H7N9 viruses that were identified while controlling the spread of the disease. It was summarized and discussed from the perspectives of molecular epidemiology, clinical features, virulence and pathogenesis, receptor binding, T-cell responses, monoclonal antibody development, vaccine development, and disease burden. These data provide tools for minimizing the future threat of H7N9 and other emerging and re-emerging viruses, such as SARS-CoV-2.

## Introduction

For decades, avian influenza viruses (AIVs) have been recorded to cross species barriers and infect humans. Before 2013, the recognized H7 subtype avian influenza virus (AIV) to cause outbreaks in humans was the H7N7 virus, which caused 89 cases of influenza in the Netherlands in 2003 [1]. Almost 10 years later in March 2013, a novel reassortant avian influenza A (H7N9) virus first emerged in the Shanghai and Anhui Province of China [2]. Since then, this virus has spread from eastern to southern and northern China; thus far, 1568 laboratory-confirmed cases of human infection with H7N9 viruses with a mortality rate of 39.2% have been reported to the World Health Organization (WHO) [3,4].

The emergence of H7N9 viruses seems to have opened Pandora's box. During the first four waves of the H7N9 influenza epidemic, the H7N9 viruses that circulated among poultry and occasionally caused human cases in China were classified as low-pathogenic (LP) AIVs (LPAIVs), which caused asymptomatic infections in poultry [4,5]. New isolates emerged later, possessing a cleavage site of polybasic amino acids originating from the insertion of four amino acids into the hemagglutinin (HA) protein [6,7] and exhibiting high pathogenicity to chickens during wave 5 from 2016 to 2017 [8–10]. Several outbreaks of these highly pathogenic (HP) AIVs (HPAIVs) on poultry farms have resulted in the deaths of approximately 110 000 birds across 10 provinces [11] and a series of human cases.

A series of public health intervention strategies involving different institutes under the Task Force of Joint Prevention and Control System in China have responded well to the outbreak and controlled the epidemic [12]. Several studies have shown that the closure of live poultry markets (LPMs) in the cities that were most affected by H7N9 epidemics was highly effective in reducing the risk of H7N9 infection in humans [13–15]. Other sustainable and effective interventions in the LPM system, including rest days and the banning of live poultry overnight, also reduced the risk of this zoonotic influenza. A bivalent inactivated H5/H7 vaccine for chickens was also introduced [16]. Since wave 5, only sporadic cases of human infections have been reported. Therefore, the multiple strategies against emerging H7N9 viruses seem to have been quite successful thus far [17]. Here, we review the current characteristics of the H7N9 viruses that were identified while controlling their spread in terms of molecular epidemiology, clinical manifestations, virulence and pathogenesis, receptor binding, antibody and T cell immunity, vaccine development, and disease burden. These data extend our understanding and reduce the current and future threats posed by H7N9 and other zoonotic viruses such as SARS-CoV-2.

## Molecular epidemiology and evolution

### Origin and genesis of H7N9 AIVs

The genesis and origin of the H7N9 viruses were inferred at the beginning of the H7N9 epidemic on the basis of the genome sequences of the H7N9 viruses and other AIVs. Genetic analyses indicated that H7N9 is a triple reassortant of the H7, N9, and H9N2 AIVs (Fig. 1A) [3,18]. The HA of H7N9 is most closely related to that of H7N3 strains isolated from Zhejiang Province, whereas N9 neuraminidase (NA) may have originated from the H7N9 viruses identified in ducks and wild birds in Korea. Long-term surveillance data for domestic poultry suggested that the two segments were transferred from wild birds to domestic ducks along the East Asian flyway and then to chickens in LPMs (Fig. 1A) [3,19–21]. One type of enzootic H9N2 virus that has become predominant in chickens since 2010 provided all six internal genes and established the novel H7N9 virus by reassortment (Fig. 1A) [22]. Further analysis found that reassortment events likely occurred in the Yangtze River Delta around 2011–2012 [3,20]. According to an in-depth evolutionary analysis of genomic sequences, the H7N9 viruses may have been generated via at least two steps of sequential reassortment involving distinct H9N2 donor viruses in different hosts [23]. Wu *et al.* suggested that the first reassortment event likely occurred in wild birds and generated the SH/1-like viruses (A/Shanghai/1/2013(H7N9)). In the second event, the reassortment of SH/1-like viruses and A/chicken/Jiangsu/ZJ4/2013(H9N2)-like viruses led to the emergence of the diverse genotypes of the H7N9 viruses in China. Although the genesis and origin of H7N9 have been inferred, the details of the precise events remained unclear because at that time, surveillance data for domestic/wild birds were still scant.

### Evolution of H7N9 AIVs since 2013

The H7N9 viruses that circulated in domestic poultry in China have caused five epidemic waves of human infections and evolved continuously and substantially since their emergence in 2013 [3,8,20,24–26].

Across all five epidemic waves of human infections (2013–2017), the HA and NA genes of the H7N9 viruses have shown sequential evolution insofar as the surface proteins of the isolates in the later waves were derived from those in the earlier waves [19,27]. The H7N9 viruses of wave 1 were mainly from the Yangtze River Delta region and had similar surface proteins, suggesting that these viruses had a common source [20,24,25,28]. The H7N9 viruses of wave 2 were derived from those of wave 1, and the first re-emergent H7N9 virus of wave 2 was very similar to the viruses of the first wave [24,29]. In wave 2, the viruses dispersed from the Yangtze River Delta region to the Pearl River Delta region; they circulated in both regions and formed two regional lineages (Fig. 1B). The viruses of wave 3 were phylogenetic related to those in wave 2, and the majority of viruses of wave 3 were detected in the Pearl River Delta region (Fig. 1B) [24,25]. The viruses of waves 4 and 5 were mixed and clustered together, thus showing a genetically close relationship (Fig. 1B). The viruses of wave 4 mainly circulated in the Pearl River Delta region, whereas those of wave 5 circulated in the Yangtze River Delta region and the Pearl River Delta region (Fig. 1B). Phylogenetic analysis showed that the genetic diversity and population size of the H7N9 viruses increased across the five epidemic waves. In the first wave, H7N9 displayed low nucleotide diversity because it had insufficient time to accumulate mutations [30]. After the first outbreak, H7N9 experienced rapid population expansion, which was accompanied by its dissemination from the Yangtze River Delta to the Pearl River Delta region [24,30].

Historically, among all 16 subtypes of AIVs, only H5 and H7 possess HA that has acquired basic amino acids at the cleavage site. This phenomenon converted these viruses from LP to HP. Unsurprisingly, this conversion also occurred in H7N9. In the first four epidemic waves, the H7N9 AIVs were classified as LPAIVs. During December 2016 and January 2017, some H7N9 viruses with a multiple basic motif (PKRKRTA(R/G), PKGKRTA (R/G), or PKGKRIA(R/G)) at the cleavage site of the HA

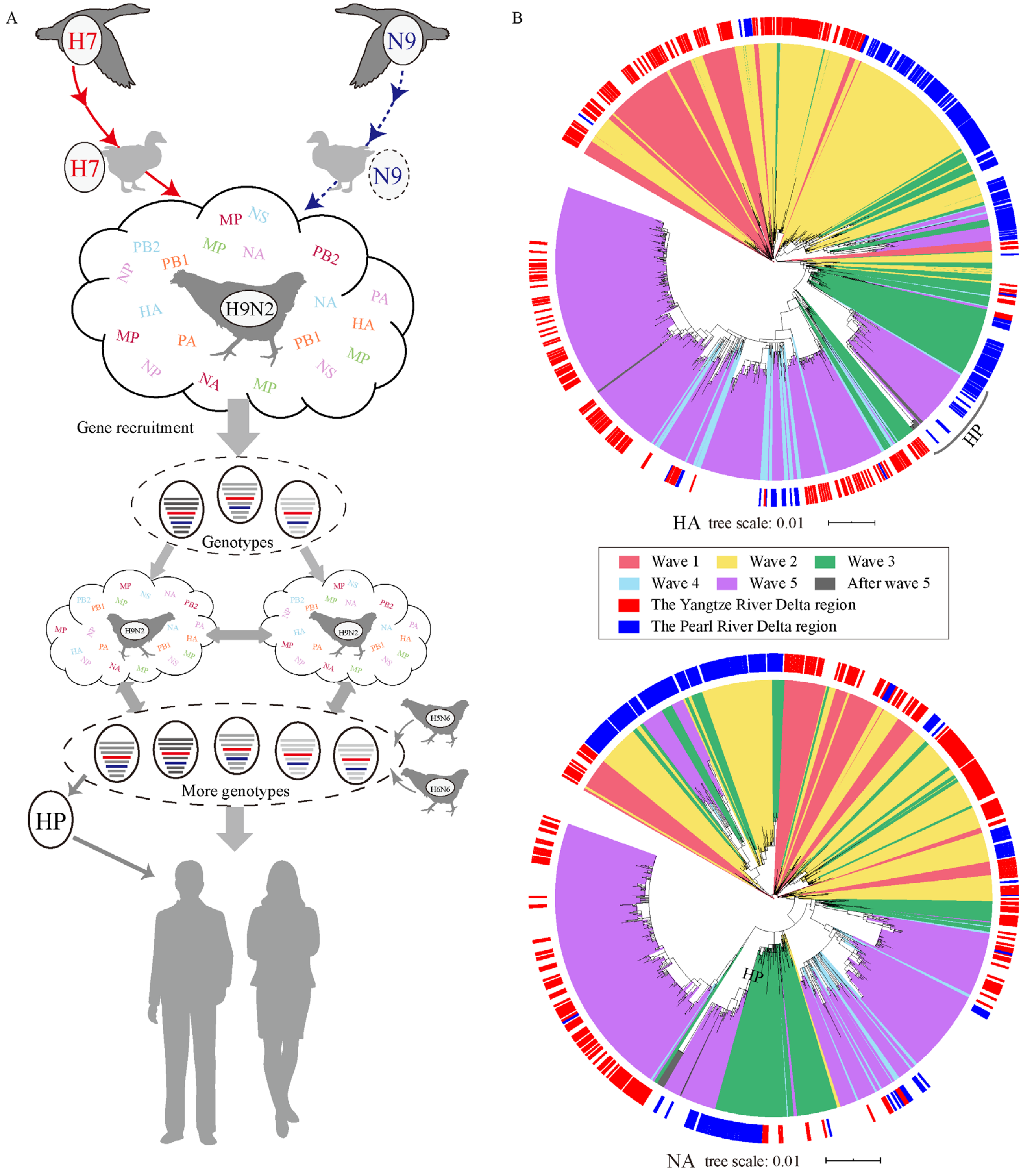

**Fig. 1** Origin and evolution of H7N9 viruses. (A) Schematic of the origin and dynamic reassortment of H7N9 viruses. The migratory birds, ducks, chickens, and humans involved in the emergence and outbreaks of H7N9 are shown. The dashed line arrow and circle of N9 represent the uncertain presence of N9 in ducks as indicated by a previous study [19]. Colored internal gene names in clouds represent diversified H9N2 gene pools. The specific reassortment of H7N9 with the H5N6 and H6N6 viruses is also shown. HP represents the highly pathogenic H7N9 viruses, which have also caused human infections. (B) Maximum likelihood (ML) trees of the surface genes of the H7N9 viruses in all human infections. Sequences of the surface genes of the H7N9 viruses in all human infections were downloaded from the NCBI Influenza Virus Database and Global Initiative on Sharing All Influenza Data for phylogenetic analysis. ML trees were inferred with the software RAxML under the GTRGAMMA model with 1000 bootstrap replicates by using A/Shanghai/02/2013 as the root. The background colors of the branches show the time at which the corresponding virus was isolated and are classified as waves 1 to 5 and after wave 5. Colored strips far from the tree indicate the regions from which the corresponding viruses were isolated. The Yangtze River Delta region, the Pearl River Delta region, and other regions are labeled in red, blue, and no-color, respectively. HP in the upper panel shows the cluster containing all HP H7N9 isolates.

protein were identified as HP in poultry [6,8,31]. The HA and NA genes of the HP-H7N9 viruses have common ancestors (Fig. 1B) [8].

The H7N9 viruses have not only established regionally distinct lineages but have also greatly increased in genotypic diversity through internal gene reassortment [30]. Across the five epidemic waves of H7N9 viruses, their internal genes have undergone complex reassortment with those of different AIVs, especially with those of the H9N2 viruses in poultry. In the first wave, high genetic heterogeneity was observed in internal genes, and at least 26 genotypes were identified [19,32]. A dynamic reassortment model was used to explain the evolution of the H7N9 viruses in the first wave (Fig. 1A) [19]. During the first wave, these viruses entered enzootic AIV hosts and began to recruit internal genes to adapt themselves to their new hosts [19]. An analysis of the first-wave viruses from humans and evidence from animal models revealed that continuous reassortment and amino acid substitutions, such as at residue 627 of polymerase basic 2 (PB2), mediated the adaptation of the H7N9 viruses to humans through their interspecies transmission [32,33]. During waves 2 and 3, the internal genes were further divided into various clades [25]. Genetic heterogeneity increased, leading to the creation of 93 genotypes, although most were transient [25]. This dynamic reassortment has resulted in the abundant genotypes of the H7N9 viruses. A recent study provided evidence for the reassortment of H7N9 with the H5N6 and H6N6 viruses, suggesting that the H7N9 viruses had a larger gene pool than that previously thought when they recruited these internal genes [34].

## Clinical features

As infection by other severe viral pneumonias, most H7N9-infected patients develop pneumonia that does not respond to broad-spectrum antibiotics directed against typical and atypical bacteria [35]. The pneumonia typically extends to both lungs, and the patient deteriorates rapidly. Fever and coughing are the most common symptoms together with sputum production, dyspnea, and hemoptysis [36]. Patients infected with H7N9 have other nonspecific symptoms, including headaches, fatigue, and myalgia. Chest radiography or computed tomography scans usually show bilateral ground-glass opacities and consolidation, which are typical characteristics of viral pneumonia (Fig. 2). Other radiographic features include interlobular septal thickening, centrilobular nodules, reticulation, cystic changes, bronchial dilatation, subpleural linear opacities, and pleural effusion [37–39]. In moribund patients, the common outcomes are acute respiratory distress syndrome, severe pneumonia, multiple organ failure, and death at 8–43 days after symptom onset [9,36,37,40].

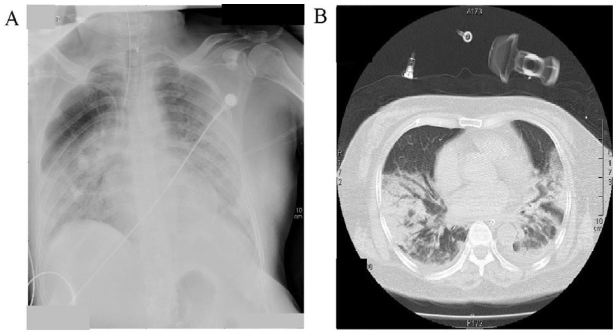

**Fig. 2** Radiographic findings in A (H7N9) pneumonia. Chest radiograph (A) and computed tomography scan (B) showing bilateral ground-glass opacities and consolidation.

Laboratory tests showed lymphopenia and thrombocytopenia in over 88% and 73% of patients, respectively [36]. Severe H7N9 infection is associated with elevated levels of C-reactive protein, serum creatinine, and myoglobin and with T cell lymphopenia (Table 1) [41]. Many patients experience nosocomial bacteremia and bacterial pneumonia, including infections with *Acinetobacter baumannii*, *Klebsiella pneumonia*, and *Pseudomonas aeruginosa* [39,42,43]. Given that the majority of the patients have already received broad-spectrum antibiotics, most of the organisms causing these nosocomial infections are resistant to multiple antibiotics [42].

During wave 5, which started in October 2016, 766 laboratory-confirmed cases and 288 deaths (case fatality rate (CFR): ~37.6%) were recorded in China by the end of September, 2017. The HPAIV H7N9 variant that emerged in cases during wave 5 has been identified as the causative pathogen of several human infections [6,8]. Human cases of infection with HP-H7N9 AIVs were identified in Guangdong, Guangxi, Fujian, Hunan, Shaanxi, Hebei, Henan, and Yunnan provinces [6,27,44]. However, several other provinces, such as Heilongjiang, Inner Mongolia, Shandong, Beijing, and Tianjin, have also reported HP-H7N9 outbreaks in poultry or its detection in LPMs. Notably, H7N9 infections in several family clusters and in patients in the same hospital wards were also reported.

Although the greatest numbers of human cases occurred in wave 5 [7,45], current evidence indicates that the newly emergent HP-H7N9 causes a disease in humans with a severity similar to the severity of LP-H7N9. A statistical analysis of several cases in Shenzhen, China, identified no significant differences in patients infected with HP- or LP-H7N9 in terms of age, days from onset to hospital admission, coexisting chronic medical conditions, and complication incidence during hospitalization. Patients in both groups also showed similar patterns of $PaO_2/FiO_2$ dynamics and imaging characteristics associated with pathological changes (Table 1) [37]. The overall case fatality rate (CFR) in Shenzhen was approximately 13.3%,

**Table 1** Clinical characteristics and laboratory results of subjects hospitalized for infection with low pathogenic and highly pathogenic H7N9 during the five waves of the H7N9 epidemic in China

| Parameter[a] | HP (wave five, $N$ = 5)[b,c] | LP (wave five, $N$ = 7) | $P$ value[e] | LP (first four waves, $n$ = 123)[d] | $P$ value[e] |
|---|---|---|---|---|---|
| WBC ($\times 10^9$/L)[f] | 5.02 (4.81–5.6) | 3.88 (3.52–6.39) | NA[g] | 4.5 (2.9–6.2) | NA |
| LYM ($\times 10^9$/L) | 0.505 (0.48–0.56) | 0.53 (0.47–0.86) | NA | 0.5 (0.3–0.7) | NA |
| NEU ($\times 10^9$/L) | 4.18 (4.13–4.73) | 3.22 (3.1–4.97) | NA | 3.3 (2.2–5.4) | NA |
| PLT ($\times 10^9$/L) | 166 (160–221.5) | 157 (139–198.3) | NA | 114 (82–147.5) | NA |
| AST (U/L) | 64.2 (38.5–107.4) | 88.3 (42.85–224.9) | NA | 53 (38–96.5) | NA |
| ALT (U/L) | 63.7 (40.4–112.4) | 74 (44.05–119.7) | NA | 35.5 (24–64.5) | NA |
| CRE (μmol/L) | 60 (53–107.15) | 89.65 (62.1–106.6) | NA | 70.7 (58.3–85) | NA |
| CK (U/L) | 182 (144–239.5) | 163.8 (123.75–354) | NA | 195 (96–562) | NA |
| CRP (nmol/L) | 92.65 (74.6–176.2) | 72.5 (49.7–83.3) | NA | 65 (25–113) | NA |
| ALB (g/L) | 31.9 (26.3–32.5) | 32.6 (32.5–33.7) | NA | NA | NA |
| LDH (U/L) | 711.5 (573.8–848.5) | 994.5(475.8–1596.5) | NA | 498 (388–661) | NA |
| Leukopenia | 0/4 (0%) | 4/7 (57%) | 0.194 | 48/105 (46%) | 0.129 |
| Lymphopenia | 4/4 (100%) | 5/5 (100%) | NA | 88/99 (89%) | 1.000 |
| Neutropenia | 0/4 (0%) | 0/6 (0%) | NA | 13/103 (13%) | 1.000 |
| Neutrophilia | 0/4 (0%) | 0/6 (0%) | NA | 5/103 (5%) | 1.000 |
| Thrombocytopenia | 0/3 (0%) | 3/6 (50%) | 0.464 | 80/104 (77%) | 0.015 |
| Elevated AST | 2/4 (50%) | 4/7 (57%) | 1.000 | 54/103 (52%) | 1.000 |
| Elevated ALT | 2/3 (66.7%) | 4/6 (66.7%) | 1.000 | 34/100 (34%) | 0.279 |
| Elevated CRE | 1/3 (33.3%) | 1/6 (16.7%) | 1.000 | 11/103 (11%) | 0.305 |
| Elevated CK | 2/4 (50%) | 2/6 (33.3%) | 1.000 | 48/98 (49%) | 1.000 |
| Elevated CRP | 4/4 (100%) | 5/5 (100%) | NA | 83/92 (90%) | 1.000 |
| Elevated LDH | 4/4 (100%) | 6/6 (100%) | NA | 89/98 (91%) | 1.000 |

[a]Results were obtained from patients at the earliest available time point after hospitalization. [b]Clinical data from two HP-H7N9 human cases are presented in this study. In addition, three cases from previous reports were included in this analysis. [c]Reference group. [d]Clinical data of the 123 cases from previous report were included in this analysis. [e]A $P$ value between 0.01 and 0.05, 0.001 and 0.01, and below 0.001 was considered statistically significant, very significant, and extremely significant, respectively. [f]Values shown represent the mean and interquartile range. [g]NA: not available.

which is far lower than the nationwide CFR during the five waves of human H7N9 infections (39.6%) [7,46]. This result may be attributable to several comprehensive measures taken, including the reasonable use of NA inhibitors, mechanical ventilation, corticosteroids, antibiotics, and fluid infusion, and the strict prevention and control of nosocomial infections. However, this difference requires further confirmation because the sample sizes in the studies were limited.

Despite the absence of evidence showing that HP-H7N9 is more virulent than LP-H7N9 in humans or more transmissible as a consequence of its increased infectivity of the respiratory epithelium in the nasal passages [6,47], the pathogenesis of HP-H7N9 in poultry and its resistance to NA inhibitors (NAIs) may still affect its human infection characteristics. The case numbers showed that in the first four waves, patients infected with HP-H7N9 had a higher CFR (14/28, 50%) than those infected with LP-H7N9 (324/798, 40.6%) [37,45]. Poultry infected with HP-H7N9 become sick and may shed high concentrations of the virus, increasing the risk of human infection. The emergence of antiviral resistance is associated with adverse clinical outcomes for human H7N9 infections [47]. The overall incidence rate of NAI resistance in HP-H7N9 carrying the mutation R292K is approximately 28% (13/46), which is far higher than that in LP-H7N9 (34/1225, 2.8%) [37]. This characteristic suggests that HP-H7N9 may develop the NAI-resistance mutation easily during antiviral treatments with NAIs. This situation may be one reason for the higher CFR of HP-H7N9 [37]. Therefore, the NAI-resistance mutation should be closely monitored during the treatment of patients infected with HP-H7N9.

## Virulence and pathogenesis

When H7N9 emerged in 2013 [48], the LP-H7N9-infected chickens lacked obvious clinical symptoms, and the virus mainly replicated in the upper respiratory tract [49,50]. However, LP-H7N9 caused severe disease in humans in the first four waves of the epidemic [5]. Over 85% of H7N9-infected patients had a history of exposure to poultry. An HP-H7N9 mutant in which the insertion of four amino acids generated multiple basic amino acids at the cleavage site of the HA protein emerged during epidemic wave 5 [6,7]. This mutant caused severe disease and outbreaks in poultry [8,51]. Importantly, a study showed that one HP-H7N9 strain was more pathogenic to ferrets than LP-H7N9 with evidence of its effective transmission via respiratory droplets [52]. Another study showed that although HP-H7N9 isolates were more virulent than LP-H7N9 viruses in mouse and ferret animal models, these viruses were poorly transmissible via respiratory droplets [53]. However, the virulence and transmissibility of an HP-H7N9 strain substantially increased in mammals after adaptation during one passage in ferrets [54]. In fact, different epidemic H7N9 strains, including LP-H7N9 and HP-H7N9, displayed diverse virulence and transmissibility in mammals, and the public risk of H7N9 did not increase during wave 5 [55]. As discussed in the clinical section above, the current evidence does not support the notion that HP-H7N9 is more virulent or transmissible than LP-H7N9 in humans.

The contributions of the six internal genes of H7N9 (A/Anhui/1/2013) to its virulence in mice were evaluated on the basis of the H9N2 backbone. The PB2, matrix (M), and nucleoprotein (NP) genes were identified as the virulence genes necessary for high pathogenicity in mice. In particular, the gene segments from H9N2 that recombined with the H7N9 PB2 gene caused more severe disease in mice with higher mortality rates and cytokine secretion than the parent H7N9 (A/Anhui/1/2013) and other H9N2 recombinants that included different internal H7N9 genes [33]. The E627K mutation in PB2 was identified as the critical mutation and conferred the high virulence of H7N9 in mice (Table 2). This critical mutation emerged as early as 4 days after infection [33]. In addition to E627K, other mutations, such as T271A, Q591K, D701N, A588V, and K526R, in PB2 contribute to the pathogenicity and replication capacity of H7N9 in mammals and/or mammalian cells (Table 2) [56–58]. Notably, we observed the gradual replacement of 627E by 627K in H7N9, termed "genetic tuning" in the longitudinally collected specimens from infected patient and the correlation between rapid host adaptation of H7N9 PB2-E627K and the fatal outcome and disease severity in humans [59]. Furthermore, a long deletion (10–20 amino acid residues) in the stalk of NA increased the pathogenicity of H7N9 in mice (Table 2) [60], although no wild-type viruses with these deletions have been detected. Therefore, the mutation and evolution of H7N9 AIVs and their virulence and pathogenesis must be closely monitored. So that necessary measures can be timely implemented to control the risks posed by potential mutants.

## Distinct avian-to-human receptor binding adaptation of H7

The receptor binding property of the HA protein is one of the crucial determinants of the interspecies transmission of influenza viruses. In the first step of viral entry, influenza viruses must be able to bind sialic acid (SA) receptors on the host cells to allow host jumping [61–65]. Typically,

**Table 2** Critical amino acid residues in H7N9 proteins associated with viral virulence in mammals

| Protein | Amino acid position | Potential biological functions |
|---|---|---|
| HA[a] | S138A | Responsible for the acquisition of human receptor binding capacity [70] |
| | T221P | |
| | G186V | Responsible for the acquisition of human receptor binding capacity [70,72] |
| | Q226L | Critical for binding the α-2,6-linked receptor and enables transmission in mammals [70,72,174,175] |
| | Insert-KRTA-at the HA cleavage site | Contributes to disease in mice [176] |
| | HA2-K64E | Reduces viral stability and replication in mice [176] |
| NA | 19- to 20-amino-acid deletion in the NA stalk | Enhances virulence in mice [60] |
| PB2 | T271A | Enhances viral replication in mammalian cells *in vitro* [175] |
| | K526R | Enhances viral replication in mammalian cells and in mice [56] |
| | A558V | Promotes mammalian adaptation [58] |
| | E627K | Associates with increased virulence of AIVs in mammals [33,175,177–179] |
| | Q591K | Increases pathogenicity in mice [177,178] |
| | D701N | |
| NP | V41I and/or D210E | Promotes the replication capability of H7N9 viruses at low temperature and thus might contribute to viral transmissibility [180] |
| | A286V | Attenuates the virulence of H7N9 viruses in mice [181] |
| | T437M | |
| NS1 | V178I | Promotes viral replication in mice [182] |
| | P212S | |

[a]H3 numbering.

AIVs preferentially bind the α-2,3-linked SA glycan receptor (avian-type receptor), whereas human influenza viruses preferentially bind α-2,6-linked SA glycan receptors (human-type receptor). Human-infecting AIVs are indicated to have originated from strains that have gradually evolved the capacity to bind human-type receptors [66].

The receptor binding site (RBS) of the HA protein consists of three critical secondary elements: the 130-loop, 190-helix, and 220-loop. The key interacting residues within each element display remarkable variations among different influenza virus subtypes; these variations result in distinct receptor binding properties [63]. The key determinant residues for human receptor binding or a human/avian receptor preference can be significantly distinct in different HA-subtype influenza viruses. A certain HA subtype can evolve from avian receptor specificity to dual-receptor tropism or even to human receptor preference via more than one pathway [67]. Through biochemical and structural studies, we have gained a substantial understanding of the determinants of the receptor binding properties of the H7 subtype influenza viruses that have caused human infections.

The H7N9 outbreak in 2013 and the more recent H7N4 human infections have highlighted the potential of the H7 subtype AIVs to adapt to human hosts and evolve into human influenza viruses [4,68,69]. Although previous studies have shown that H7N9 subtype influenza viruses have not acquired the capacity for efficient human-to-human transmission, an increase in the human-receptor binding capacity of H7 subtype AIVs was observed before and after the 2013 outbreak [70]. Based on our current knowledge, the receptor binding property of the H7 subtype HA is mainly determined by residues at two positions in RBS, i.e., residues 186 and 226 (Fig. 3) [71–73]. Two other sites, namely, residues 138 and 221, also play a cooperative role in viral binding affinity [70]. In contrast to the human-receptor binding capacity of the other HA subtypes, including the H2, H3, H4, and H5 subtypes, canonical signatures (such as the Q226L substitution) are not the key to the human-receptor binding capacity of the H7 subtype HA. Instead, a single G186V substitution determines the human-receptor binding capacity of avian H7 subtype HA and may have emerged much earlier in the evolutionary process than other substitutions [70] (Fig. 3). By contrast, the Q226L substitution may have occurred later than other substitutions to further regulate viral affinity or preference for human-type receptors. Importantly, L226 only favors human- and avian-type receptors when paired with hydrophobic

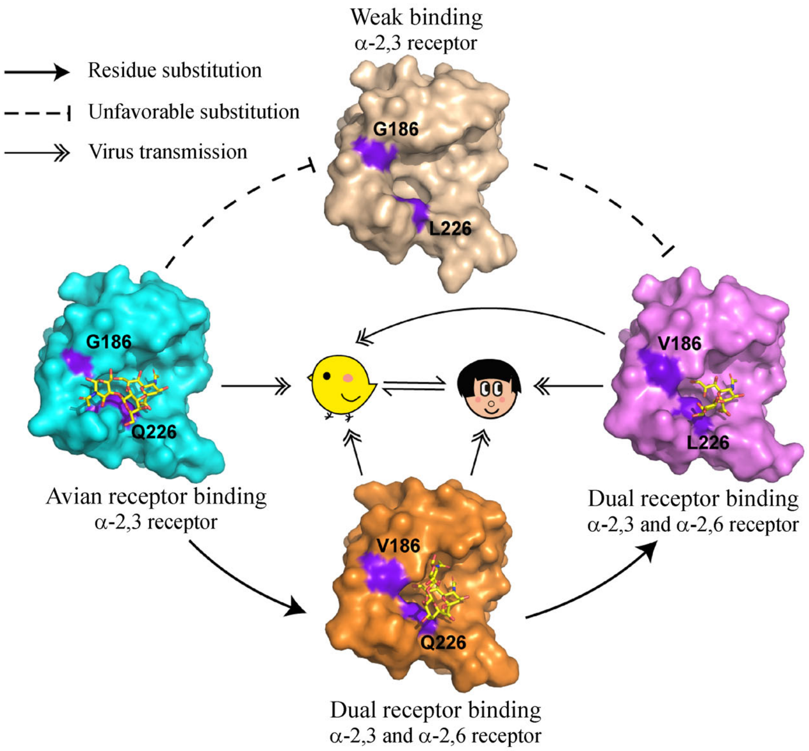

**Fig. 3** Diagram of the evolutionary routes of H7N9 HA. H7 HA with different residue combinations at receptor binding sites (G186/Q226, V186/L226, G186/L226, and V186/Q226) are represented by surface sheets and colored cyan, violet, wheat, and orange. SA receptor analogs are shown as sticks and colored on the basis of elements (carbon, yellow; oxygen, red; nitrogen, blue). The positions of residues 186 and 226 are highlighted in purple.

residues at positions 138 and 186 [70]. Structural evidence indicates that the G186V substitution changes the local hydrophobicity of RBS, thus affecting the conformation of the side chain of residue E190, which directly interacts with the SA receptor [70]. However, the Q226L substitution alters the stability of the 220-loop, affecting viral binding to human- and avian-type receptors [70]. Bioinformatic analyses have shown that the circulating H7 subtype influenza viruses are mainly dual-receptor tropic with a preference for human receptors [7,70]. Artificial H7 protein mutants with human receptor specificity have also been developed in the laboratory [74]. These mutants may be used to reveal the risk of the further adaptation of the H7 subtype influenza viruses.

## Cell-mediated immunity

### Cell-mediated immunity in the acute phase of H7N9 infection

Infection with the H7N9 influenza virus is characterized by high inflammatory cytokine and chemokine levels in the lungs and peripheral blood; this condition is called hypercytokinemia or cytokine storm [75–77] and is thought to be correlated with severe disease in patients with H7N9. However, the hypercytokinemia levels caused by H7N9 are lower than those caused by H5N6 [76,78–82]. A comparative study showed that the concentrations of some cytokines induced by HP-H7N9 in human patients seemed higher than those induced by LP-H7N9, although the differences were not significant [37].

A dramatic increase in lymphocyte subgroups was observed in the early stage of H7N9 infection in patients who survived. This increase led to significantly elevated levels of most lymphocyte subgroups during the recovery phase. By contrast, in patients who succumbed to infection, the T cell population remained at a low level during hospitalization and decreased suddenly on the day of death [83]. In the acute phase, the expression of human leucocyte antigen (HLA)-DR on $CD14^+$ cells was negatively correlated with the severity of H7N9 infection and might result in impaired T cell response due to low antigen-presenting capacity [84]. Patients with severe H7N9 presented with a delayed T cell response in the acute phase. However, robust early $CD8^+$ T cell responses in patients were correlated with rapid recovery from the disease [85]. The early transient prevalence of highly activated $CD38^+HLA\text{-}DR^+PD\text{-}1^+CD8^+$ T cells was observed in surviving patients, whereas the prolonged persistence of this cell population was observed in ultimately fatal cases [85]. The differential clonal expansion kinetics of T cell receptor $\alpha\beta$ were observed between the surviving patients infected with H7N9 and those who died [86]. Deep-sequencing the transcriptomes of T and B cells from H7N9-infected patients showed higher B cell diversity but lower T cell diversity in surviving patients than in those who died [87]. The studies on the immune status of hospitalized patients suggested that T cells play a pivotal role in immune protection against H7N9 during the acute phase of the disease.

### Cell-mediated immunity in convalescence

A series of studies on the immunology of H7N9 survivors during follow-up identified uncommon characteristics of the survivors' T cell responses after severe AIV infection. A 1-year follow-up of survivors showed that the total lymphocyte count had normalized by 1 month postinfection [88]. However, evidence for the ongoing impairment of the immune responses in patients who had suffered severe infections was found; this evidence included reduced HLA-DR expression by $CD14^+$ monocytes and reduced interferon-$\gamma$ (IFN-$\gamma$) production by T cells. During nonspecific stimulation with *Streptococcus* antigens, T cell responses were abnormal in severely affected patients 1 month after infection compared with those in patients with mild symptoms. The abnormal immune status of the general T cell population returned to normal by 3 months postinfection [88]. In a cohort of H7N9 survivors who were followed up for 15 months after infection, although the H7N9-specific antibody concentrations declined over time, the frequency of virus-specific IFN-$\gamma$-secreting T cells increased within 1 year of infection [89]. This phenomenon was confirmed in another cross-sectional study involving survivors at 36 months after infection [90]. This increasing trend over time within 1 year of infection was more obvious in patients aged $\geqslant$ 60 years and in critically ill patients requiring ventilation during their infections. This trend was attributed to the initially considerably lower percentage of H7N9-specific IFN-$\gamma$-secreting T cells within 4 months of infection in these patients than in younger and more mildly affected patients. The levels of antigen-specific $CD8^+$ T cells expressing the lung homing marker CD49a in samples obtained at 6–8 months after H7N9 infection were higher than in those obtained at 1.5–4 months after infection [89]. In our study on survivors who were followed up for 36 months, the percentage of virus-specific cytokine-secreting memory $CD8^+$ T cells peaked in the survivors at 14 months after infection and declined gradually thereafter [89]. A linear regression analysis showed that underlying medical conditions, lack of antiviral therapy, and female sex were predictors of higher T cell responses in the survivors. The prolonged reconstruction and evolution of virus-specific T cell immunity in survivors of H7N9 infection showed new immune features directed against severe AIV infection, which may have implications for T cell-directed immunization strategies.

### Cross-reactive T cell immunity between H7N9 and other influenza viruses

The envelope proteins of the H7N9 virus, such as HA and NA, are highly variable. By contrast, the internal proteins, such as matrix 1 (M1), NP, and polymerase basic 1 (PB1), of H7N9 share highly conserved sequences with those of other subtypes of the influenza A virus (IAV). A series of comparable studies showed that a strong cross-reactive T cell response was induced by seasonal IAVs and H7N9 [91,92] despite substitutions in the T cell epitopes. $CD8^{+}$ T cells that cross-reacted with H7N9 viruses were already present in the peripheral blood of a healthy European population before the H7N9 epidemic. In particular, the H7N9 variant of the $NP_{418–426}$ epitope was recognized by seasonal H3N2-specific $CD8^{+}$ T cells derived from an HLA-B*35 subject [91]. Quinones-Parra *et al.* also showed that antigenic peptides derived from H7N9 were recognized by memory $CD8^{+}$ T cells that were generated during previous influenza exposure but that the capacity for recognition varied among different ethnicities with different diversities of HLA alleles [93]. As indicated by our previous study, cross-reactivity between different influenza viruses may be attributable to immunodominant T cell epitopes [94]. In a recent study, we found that although 2009 pandemic H1N1 (pH1N1)-specific T cells showed biased reactivity to human-infecting H5N1, the pre-exposure of mice to H1N1 provided protection against H5N1 and H7N9 challenge [95]. This result was confirmed in other studies on mice wherein cross-reactive memory T cells offered heterosubtypic protection against H7N9 [96,97]. Therefore, the pre-existing cross-reactive T cell immunity induced by previous infection with the seasonal influenza virus in the population may contribute to the clearance of H7N9 and symptom relief in patients [4].

Although broadly cross-reactive T cell immune responses are induced by H7N9 and other seasonal influenza viruses, H7N9-virus-specific substitutions in dominant T cell epitopes can lead to immune evasion in subjects naïve to H7N9 [92,93,95]. We previously demonstrated that two amino acid substitutions in an HLA-A*1101-restricted peptide, H1-P22 ($NP_{188–198}$: TIA-MELIRMIK in pH1N1), led to dramatic antigenic variability in the corresponding T cell epitope H7-22 (TMVMELIRMIK) in H7N9 (Fig. 4A–4C) [92]. Another M1-protein-derived peptide, H1-P25 ($M1_{99–109}$: LYKKLKREITF in pH1N1), with a HLA-A*2402 restriction has a dominant mutation with a substitution at position nine from isoleucine (Ile) to methionine (Met) in peptide H7-P25 (LYKKLKREMTF) of H7N9. This substitution does not alter the binding affinity to HLA-A*2402 but alleviates T cell recognition in HLA-A*2402$^{+}$ individuals. The crystal structures of HLA-A*2402 when complexed with peptide H1-P25 or H7-P25 (Fig. 4D–4F) show that residue Ile9 of H1-P25 inserts into the E pocket of the HLA-A*2402 groove, whereas the side chain of the mutated residue Met9 of H7-25 protrudes from the E pocket and may come into contact with TCR [95]. Similarly, the substitutions in the HLA-A*0101-restricted T cell epitope $NP_{44–52}$ of H7N9 made the peptide–HLA complex less stable and less accessible to cytotoxic T cells compared with corresponding peptide from seasonal influenza viruses [93]. A combination of structural and functional studies have demonstrated that immunogenic variations are largely modulated by substitutions in H7N9, thus affecting HLA binding and/or TCR recognition.

## Therapeutic monoclonal antibodies against H7N9 influenza virus

### H7 head-reactive monoclonal antibodies

Broadly neutralizing monoclonal antibodies (bnMAbs) are becoming a promising solution to accommodate the antigenic changes and diversity in influenza viruses. Two classes of bnMAbs have been identified: one that targets the HA head and another that recognizes the HA stem region. The membrane-distal globular head of HA is highly immunogenic and is the main target of the monoclonal antibodies (MAbs) induced during viral infection or vaccination. Thus far, several H7 HA head-targeting therapeutic MAbs have been identified in H7N9-infected patients, H7N9-vaccinated individuals, and H7-HA-vaccinated mice [98–108]. Most H7 HA head-targeting MAbs recognize the RBS and its adjacent areas [100,109]. Given that these RBS-reactive MAbs mainly function as inhibitors that block viral attachment and entry, they usually have hemagglutination inhibition (HAI) activity [98–107]. Notably, some RBS-reactive MAbs mimic the interaction of the SA receptor by directly inserting one complementarity-determining region loop into the RBS [100,109]. Some HAI antibodies, such as H7.167, bind to epitopes adjacent to the RBS of H7 HA [104].

Apart from HAI MAbs, H7 HA head side-, edge-, and bottom-reactive MAbs, such as 22-3E05, 07-5B05, and m826, have also been discovered in individuals who were administered an H7N9 vaccine or in a naïve human antibody library [103,110]. Two neutralizing MAbs, 22-3E05 and 07-5B05, derived from H7N9 vaccinees lack HAI capability but can neutralize H7N9 *in vitro* [103]. Escape mutants generated from those MAbs suggest that HA1 R65 and K182 are critical residues for the binding of 07-5B05 and 22-3E05, respectively [103]. The exact epitopes and neutralization mechanisms of these two MAbs have yet to be investigated. Another MAb, m826, was screened from a very large naïve antibody library constructed from the peripheral blood mononuclear cells of healthy adult donors. Interestingly, m826 cannot neutralize

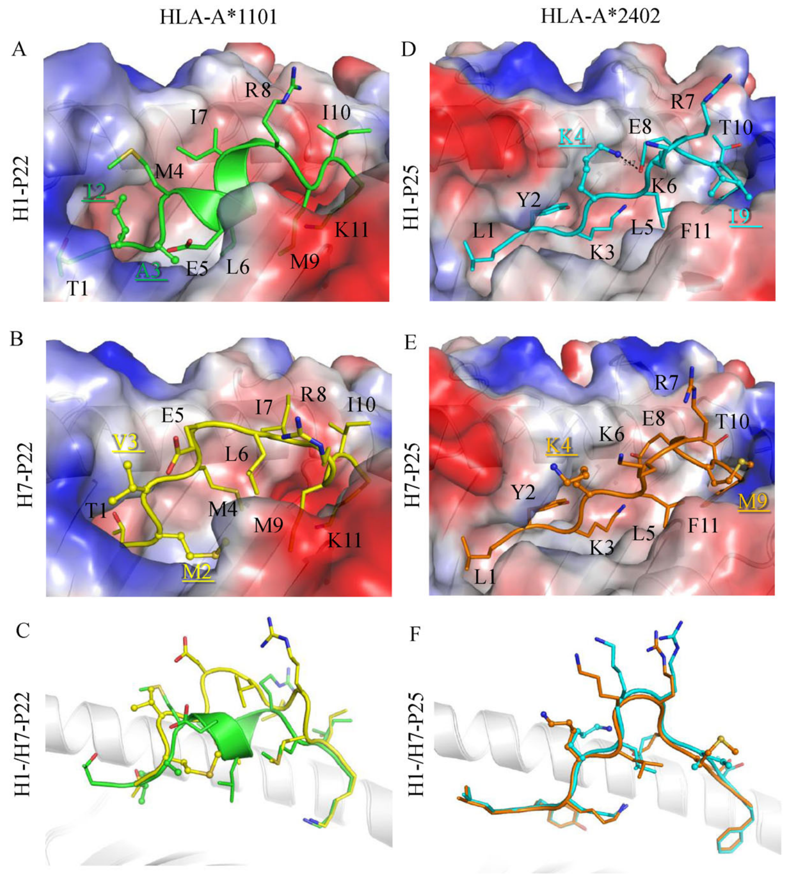

**Fig. 4** Molecular basis for cross-reactive T cell immunity and immune evasion between the H7N9 and 2009 pH1N1 influenza viruses. (A, B) 2009 pH1N1-derived T cell epitope peptide H1-P22 (PDB code: 4MJ5) and substitution peptide H7-P22 from H7N9 (PDB code: 4MJ6) presented by HLA-A*1101. The peptide-binding groove is shown as the vacuum electrostatic surface potential of the HLA-A*1101 H chain. (C) Alignment of peptides H1-P22 (green) and H7-P22 (yellow). The α1-helix of the HLA-A*1101 H chain is shown as a white spiral ribbon behind the peptide. (D, E) Peptide H1-P25 in pH1N1 (PDB code: 5WWU) and its substitution peptide in H7N9 H7-P25 (PDB code: 5WXD) presented by HLA-A*2402. The peptide binding groove is shown by the vacuum electrostatic surface potential of the HLA-A*2402 H chain. (F) Alignment of peptides H1-P25 (cyan) and H7-P25 (orange). The α1-helix of the HLA-A*2402 H chain is shown as a white spiral ribbon behind the peptide. The names and positions of the conserved residues in the peptides are denoted by black letters and numbers, respectively. Conserved residues are shown with colored sticks. Residue substitutions are shown in underlined bold text in colors corresponding to the peptides. Residue substitutions are shown with colored spheres and sticks.

the virus *in vitro*; instead, it induces very strong antibody-dependent cellular cytotoxicity (ADCC) and is highly effective against H7N9 virus infection *in vivo* [110]. Structural analysis revealed that m826 binds to HA with high pH-dependent affinity via a unique epitope that is distinct from the conventional HA head antigenic site [110]. This unique epitope may be fully exposed during pH-induced conformational changes in HA.

Most H7 head-reactive MAbs exert a narrow breadth of protection against the H7 influenza viruses because of the high level of sequence variation and antigenic changes in this domain, which has evolved to evade immune recognition. Thus, most head-reactive bnMAbs target the RBS and have a restricted recognition pattern within a subtype, such as H1-specific 5J8 and CH65 and H2-specific 8M2 [111–114]. A few head-reactive bnMAbs, such as C05, F045-92, and S139/1, show binding and neutralization activity with more than one HA subtype [115–118]. However, none of these three RBS-reactive bnMAbs binds to H7 HA. Through continuous great effort, H7/H3 head-cross-reactive MAbs W3A1 and Z1B10 have been isolated from patients who were naturally infected with H7N9 [100]. Notably, W3A1 and Z1B10 show HAI and neutralization activity against H7 and H3 IAVs. A further structural analysis should clarify the antigenic breadth and potency of these two bnMAbs. A naturally occurring human HA head-reactive bnMAb, FluA-20, which recognizes a novel epitope at the HA trimer interface, was recently reported [119]. In contrast to RBS-reactive bnMAbs, FluA-20 recognizes the HA head in nearly all subtypes of IAVs with high affinity and protects mice from infection by the H7N9 virus. The structural characterization of FluA-20 when complexed with the H1 HA-head revealed a novel epitope at the trimer interface. This novel epitope is adjacent and lateral to, but does not overlap with, the RBS and is hidden at the HA trimer interface. This configuration indicates that FluA-20 interacts with HA in a noncanonical trimeric form, thus inhibiting the cell-to-cell spread of the virus by disrupting the integrity of the HA trimer [119].

### H7 stem-reactive MAbs

The HA stem region, which contains the N- and C-terminal fragments of HA1 and the full length of HA2, is responsible for viral and cell membrane fusion. This region is most conserved among the different subtypes of IAVs. It was previously believed that only the immunodominant HA head induces neutralizing antibodies. However, the initial discovery of C179, a stem-reactive mouse MAb, ushered in a new era of bnMAbs [120]. Many stem-targeting bnMAbs have since been discovered, and several, including CR6261, CR8020, VIS410, MHAA4549A, and MEDI8852, have entered clinical trials [121]. Most stem-reactive MAbs inhibit viral replication by locking the HA trimer in a prefusion conformation, thus preventing pH-triggered conformational changes when the virus is taken up into endocytic compartments.

After the outbreak of the H7N9 avian influenza in 2013, several H7 stem-reactive MAbs were isolated from the recipients of the H7N9 vaccine and from H7N9-infected patients [100,103,122]. These newly isolated H7 stem-reactive MAbs usually cross-react with different subtypes of HA. For example, Z3A8 and Z3B2, which were isolated from H7N9-infected individuals, are cross-reactive with H7 and H3 HA but only show neutralizing activity against H3 IAVs [100]. AF4H1K1, which was isolated from an H7N9-infected patient, binds the HAs of group 2 IAVs and protects mice from challenge with H3 and H7N9 IAVs [122]. 41-5E04, a neutralizing MAb isolated from an H7N9 vaccinee [103], competes with CR9114, a well-known stem-reactive MAb [123]. Some H7 stem-reactive MAbs, such as one human MAb (huMAb), AF4H1K1, isolated from an H7N9-infected patient, show no neutralization activity *in vitro* but instead provide protection against influenza virus infection *in vivo* [105,122]. Similar results have also been found for cross-reactive MAbs, such as 41-5D06, 07-5E01, and 24-4C01, that were isolated from H7N9-vaccinated individuals [103]. These cross-reactive non-neutralizing H7-targeting MAbs may mediate the protection afforded *in vivo* by ADCC or complement-dependent cytotoxicity through Fc–Fc receptor and Fc–complement interactions.

### H7N9 NA-targeting MAbs

NA is a subdominant target of the protective immune response, and antibodies targeting NA potentially impair viral replication by inhibiting NA activity, thereby restricting viral budding and spread [124].

The first reported MAb directed against the NA of H7N9 that exerted a therapeutic effect was 3c10-3, which was generated with hybridoma technology from a mouse vaccinated with a purified inactivated H7N9/PR8 reassortant virus [125]. Epitope mapping showed that 3c10-3 binds near the NA enzyme active site, and functional studies demonstrated that 3c10-3 inhibits the enzyme activity of H7N9 NA, blocking the cell-to-cell spread of H7N9 in cell culture. 3c10-3 has similar binding affinity for wild-type H7N9 NA and a variant NA carrying the R289K mutation, which renders it resistant to the NA inhibitor oseltamivir [126]. A panel of murine N9-specific MAbs was subsequently produced from mice vaccinated with the H7N9 NA protein [127]. Some of these murine N9-reactive MAbs, including 2F6 and 10F4, are effective against oseltamivir-resistant clinical H7N9 isolates [127].

Several H7N9 N9-specific huMAbs that were isolated from two survivors of natural H7N9 infections and two H7N9 vaccinees who were administered a monovalent inactivated H7N9 vaccine formulated with adjuvant were described at the end of 2019 [128]. These huMAbs, which exhibit NA-inhibiting activity, including NA-22, NA-45, NA-73, and NA-80, bind several antigenic sites on the N9 surface of H7N9 and mainly function by blocking the egress of nascent virions from infected cells. These huMAbs provided prophylactic and therapeutic protection to H7N9-virus-infected mice, and this protection was mediated by direct virus neutralization or an Fc-region-mediated effector function [128]. Another team also reported an H7N9 N9-specific MAb, W1C7, and three broadly N1- and N9-cross-reactive NA-directed MAbs (Z1A11, Z2B3, and Z2C2) in H7N9-infected patients [129]. Among these MAbs, Z2B3 has strong NA-inhibitory activity against clade 6B.1 pH1N1 viruses [129,130]. The isolation of these N9-targeting huMAbs indicates that either H7N9 infection or immunization with a monovalent inactivated H7N9 vaccine formulated with an adjuvant can induce potent N9-specfic NA-inhibiting and protective MAbs in humans. Recently, exciting new broadly cross-reactive and protective NA-reactive huMAbs, which can bind N2 and N7 NAs, have been identified in H3N2-infected and H7N9-infected individuals but not in individuals who were administered seasonal influenza vaccines [129,131]. The current seasonal influenza vaccines rarely induce NA-reactive B cells because key NA epitopes are poorly displayed [131].

H7- and N9-reactive MAbs that are differentially cross-reactive with HA or NA subtypes compellingly suggest that influenza vaccines should be designed to optimize the immune response to HA and NA to confer broad protection against divergent IAVs.

## Development of H7N9 influenza vaccines

Vaccination is the main intervention used to counter the infection of humans by influenza viruses. The first step in the development of an H7N9 influenza vaccine is the selection of candidate vaccine viruses (CVVs) for pandemic preparedness under coordination by the WHO. Identifying these CVVs allows timely vaccine production. During the first outbreak of the H7N9 epidemic, the A/Anhui/1/2013-like virus was recommended as the CVV by the WHO in May 2013. However, during wave 5 of the H7N9 epidemic in 2016, the circulating virus reacted poorly to ferret antiserum primed with A/Anhui/1/2013 CVVs. Therefore, the WHO suggested another H7N9 strain (A/Hunan/2650/2016) that had emerged in 2016 as the CVV [132]. These two viruses are LPAIVs. An A/Guangdong/17SF003/2016-like virus was also proposed by the WHO as the CVV for HPAIVs to replace the 2013 CVV [132]. A number of H7N9 influenza vaccines have now been developed on the basis of multiple platforms, and some vaccines have advanced to clinical trials in humans (Table 3).

### Inactivated influenza vaccines

Inactivated influenza vaccines (IIVs) are produced via traditional approaches. These vaccines are generated by infectious influenza virus grown in eggs or cell cultures and then subjected to inactivation (whole-virus vaccines) or virion disruption with detergents (split vaccines) [133]. Whole-virus vaccines based on the A/Anhui/1/2013 virus, derived from Vero cell cultures or eggs, have been described. These vaccines were protective against H7N9 challenge in a mouse model without adjuvant [133–135]. Most whole-virus and split-virion IIVs have been produced from reassortant viruses to increase vaccine yield. In these viruses, the HA and NA genes are derived from the suggested CVV, whereas the remaining six genes are derived from the highly egg-adapted strain A/PR/8/34 [133,136]. The PR8 backbone can enhance the growth titer of the CVV and reduce its pathogenicity. A number of whole-virus vaccines have been tested in mice, ferrets, and nonhuman primates and been shown to provide effective protection against lethal H7N9 challenge [137–139]. Split vaccines are usually less immunogenic in ferrets than whole-virus preparations, and high vaccine doses and oil-in-water adjuvants are required [133].

### Live attenuated influenza vaccines

Live attenuated influenza vaccines (LAIVs) are believed to be more immunogenic than IIVs because of their limited viral replication to better stimulate the immune system [140,141]. LAIVs are also reported to generate virus-specific cytotoxic $CD8^+$ T lymphocytes, which confer heterosubtypic protection [142–146]. Two LAIV platforms based on the A/Leningrad/134/17/57 (H2N2) (Len17) and A/Ann Arbor/6/60 (AA) cold-adapted master donor viruses have been licensed for human use [147,148]. The AA-based LAIV was generated with reverse genetics by using HA and NA derived from the circulating influenza virus [148–150]. The Len17-based H7N9 LAIV was generated with classical reassortment techniques [133,151]. This LAIV is highly immunogenic and protects animal models against homologous and heterologous viral challenges [133].

### Virus-like particles

Virus-like particles (VLPs) are another H7N9 vaccine platform that has advanced to clinical trials in humans. VLPs are formed via a self-assembly process that incorporates viral structural proteins (HA, NA, M1, and sometimes also M2) into ~120 nm pleomorphic particles [133]. Given that they lack viral RNA, VLPs are noninfectious and therefore safer than LAIVs. The first H7N9 VLP candidate vaccine was constructed from the full-length unmodified HA and NA genes of CVV strain A/Anhui/1/2013 and the M1 gene from A/Indonesia/05/2005 (H5N1) [152]. The H7N9 VLP was produced in insect cells with a recombinant baculovirus system. A saponin-based adjuvant (ISCOMATRIX®) or the adjuvant Matrix-M1™ was used to promote the immunogenicity and protective efficacy of the vaccine [152,153]. H7N9 VLPs have also been produced through the *Agrobacterium* infiltration-based transient expression of H7 HA protein in plants. When adjuvanted, the plant-made H7 VLP vaccine induced a protective humoral immune response in mice and ferrets [154]. In summary, H7N9 VLP vaccines are highly immunogenic, safe, and dose-sparing.

## Disease burden of H7N9 infections

Human AIV infections cause considerable population morbidity with consequent healthcare and economic burdens in addition to the considerable burden of morbidity and mortality exerted by annual global epidemics of the seasonal influenza virus [155]. Studies on these disease burdens should provide evidence that can be used to plan epidemic interventions, including vaccination programs. Public-health decision-makers also require information to compare interventions such that efforts can be concentrated on interventions that are most likely to reduce morbidity and mortality [156].

During the first outbreak of H7N9 in eastern China in 2013, the disease burden was calculated on the basis of the main drivers of economic losses, especially poultry industry losses, and the burden attributable to H7N9 infection, including direct medical costs and indirect death

**Table 3** H7N9 influenza vaccines in clinical development

| Vaccine types | Vaccine manufacturer | Clinical trial registration |
|---|---|---|
| Inactivated influenza vaccines | | |
| Subunit | Novartis, Italy | NCT01928472 |
| Split virion | Sanofi Pasteur | NCT01938742 |
| Split virion | Sanofi Pasteur | NCT01942265 |
| Split virion | Sanofi Pasteur | NCT02213354 |
| Split virion | Sanofi Pasteur | NCT02921997 |
| Split virion | GSK | NCT02177734 |
| Split virion | GSK | NCT01999842 |
| Whole virus | Medigen Vacc Corp | NCT02436928 |
| Live attenuated influenza vaccines | | |
| LAIV (Len17-based)[a] | Microgen | NCT02480101 |
| Prime/boost strategies | | |
| LAIV (AA-based)/IIV (split virion)[b] | MedImmune | NCT01995695 |
| | | NCT02274545 |
| | | NCT02151344 |
| LAIV (AA-based)/IIV (not specified) | MedImmune | NCT02957656 |
| IIV(split virion)/LAIV (AA-based) | MedImmune | NCT02251288 |
| Virus-like particle (VLP) | | |
| VLP | Novavax | NCT01897701 |
| VLP | Novavax | NCT02078674 |

[a]Len17: A/Leningrad/134/17/57 (H2N2). [b]AA: A/Ann Arbor/6/60 (H2N2).

and disability costs. This calculation showed that huge losses in the poultry industry followed the closing of LPMs and poultry slaughter and reached US$ 1.24 billion in the 10 affected provinces and US$ 0.59 billion in eight adjacent unaffected provinces [157]. The direct medical costs per patient with H7N9 were highly relative to the local average income [157,158]. H7N9 has the potential for further mammalian adaptation with limited human-to-human transmission [159,160]. Therefore, estimating the impact of a worldwide H7N9 pandemic remains necessary. Recently, an agent-based simulation model of an H7N9 pandemic that incorporates demographic information, human behavior, epidemiological characteristics, and nonpharmaceutical interventions was used to simulate events that might occur in the USA [161]. The results of this model reflect the worst-case scenario if an outbreak extends.

## Perspectives

Only four sporadic cases of human H7N9 infections, including one case of H7N9 HPAIV in late March 2019, have been reported since October 2017 [162–165]. The epidemic of this devastating AIV appears to have gradually disappeared after five waves. On the basis of their experience in fighting against different emerging and re-emerging viruses, scientists in China and other countries have investigated this virus comprehensively [166]. However, many scientific questions remain to be answered. (1) Although the phylogenetic analysis of the origin of H7N9 has clearly shown that a new reassortment event occurred, when, where, and how this reassortment occurred remain unclear. (2) Although a series of bnMAbs directed against H7N9 HA or NA have been identified, whether these bnMAbs will play a role in the treatment of severe infections requires further research and clinical trials. (3) The development of a human H7N9 vaccine seems more difficult than that of a chicken vaccine. A universal vaccine based on cell-mediated immunity or other potential strategies is a tantalizing goal to protect against H7N9 and other subtypes of AIVs. (4) Whether this AIV will flare up or re-emerge in some other status is a question that is especially important under the pressure of the widespread use of the H7N9 vaccine among chickens in China. The existence of H7N9 AIVs in LPMs is still continuously reported [164,167,168]. The phylogenetically adjacent H7 viruses are not only persistently detected in wild bird surveillance programs [69,169], but have already spilled over to induce human infections [170]. All these data demonstrate the continuous threat that H7N9 viruses pose to public health [25,82,171] and even global biosecurity [172,173]. Strengthening our continued vigilance and preparedness for a potential flare-up of this virus is imperative and will provide a preeminent model for the prevention and control of pandemic-related infectious diseases such as COVID-19.

## Acknowledgements

We would like to thank Drs. Decheng Wang, Bilin Liang from CAS Key Laboratory of Special Pathogens and Biosafety, Chinese Academy of Sciences, and Dr. Ying Xu from Nanjing Agricultural University for their assistance during the preparation of this review. This study was supported by the Shenzhen Science and Technology Research and Development Project (No. JCYJ20160427151920801), Innovation Unit for Emerging and Re-emerging Virus Adaptive Evolution Study and the Applications in Disease Control (No. 2019-I2M-5-009) and National Mega-projects for Infectious Diseases (No. 2020ZX10001016-005-001). W.J.L. is supported by the Excellent Young Scientist Program of the NSFC (No. 81822040) and Beijing New-star Plan of Science and Technology (No. Z181100006218080). H.X. is supported by Tianjin Synthetic Biotechnology Innovation Capacity Improvement Project (No. TSBICIP-KJGG-004-04). G.F.G. is a leading principal investigator of the National Natural Science Foundation of China Innovative Research Group (No. 81621091).

## Compliance with ethical guidelines

William J. Liu, Haixia Xiao, Lianpan Dai, Di Liu, Jianjun Chen, Xiaopeng Qi, Yuhai Bi, Yi Shi, George F. Gao, and Yingxia Liu declare that they have no conflict of interest. This manuscript is a review article and does not involve a research protocol requiring approval by the relevant institutional review board or ethics committee.

**Open Access** This article is licensed under a Creative Commons Attribution 4.0 International License, which permits use, sharing, adaptation, distribution and reproduction in any medium or format, as long as you give appropriate credit to the original author(s) and the source, provide a link to the Creative Commons license, and indicate if changes were made.

The images or other third party material in this article are included in the article's Creative Commons license, unless indicated otherwise in a credit line to the material. If material is not included in the article's Creative Commons license and your intended use is not permitted by statutory regulation or exceeds the permitted use, you will need to obtain permission directly from the copyright holder.

To view a copy of this license, visit https://creativecommons.org/licenses/by/4.0/.
